# A new insight into the role of plasma fibrinogen in the development of metabolic syndrome from a prospective cohort study in urban Han Chinese population

**DOI:** 10.1186/s13098-015-0103-7

**Published:** 2015-12-02

**Authors:** Lijie Ding, Chengqi Zhang, Guang Zhang, Tao Zhang, Min Zhao, Xiaokang Ji, Zhongshang Yuan, Ruihong Liu, Fang Tang, Fuzhong Xue

**Affiliations:** Division of Biostatistics, School of Public Health, Shandong University, PO Box 100, Jinan, 250012 Shandong China; Health Management Center, Shandong Provincial QianFoShan Hospital, Jinan, Shandong China

**Keywords:** Fibrinogen, Metabolic syndrome, Cohort, Overweight

## Abstract

**Background:**

Elevated levels of fibrinogen may contribute to a prothrombotic state. Cross-sectional studies suggest fibrinogen possibly linked with MetS/its components, while results of cohort studies remain controversial. Thus, this study was designed to identify the association of plasma fibrinogen with metabolic syndrome (MetS) and further to clarify the role of fibrinogen in the development of MetS.

**Methods:**

A large-scale prospective cohort study was conducted in routine health check-up population. 6209 participants free of MetS at baseline were included in the original cohort, with annually routine health check-up for incident MetS from 2005 to 2011. Then, 4 pre-MetS sub-cohorts, with overweight, hypertension, hyperglycemia and dyslipidemia at baseline respectively, were also created from the original cohort. Various strategies of Cox model analysis were performed for attempting to confirm the role of fibrinogen in the development of MetS.

**Results:**

Total MetS incidence density was 75.58 per 1000 person-years. Cox regression analysis by adjusting for potential confounders as well as four MetS components showed a significant effect of fibrinogen on MetS just in female, with risk ratio (RR) (95 % CI) of 1.48 (1.02, 2.13) for Q4 vs. Q1. Further analysis in the 4 pre-MetS female sub-cohorts revealed this significant effect only in overweight sub-cohort, with RR (95 % CI) of 1.97 (1.20, 3.23), but no significant interaction of overweight with fibrinogen on MetS was revealed in original female cohort. Then, stratification analysis among the 4 sub-groups of fibrinogen quartiles showed that effects of overweight on MetS were different among the 4 sub-groups of fibrinogen quartiles, with RR of 2.98 for Q1, 4.40 for Q2, 3.93 for Q3, and 4.82 for Q4 respectively.

**Conclusions:**

Fibrinogen was associated with MetS just in overweight sub-cohort of female individuals, and fibrinogen might be a potential modifier in the pathway from overweight to MetS.

**Electronic supplementary material:**

The online version of this article (doi:10.1186/s13098-015-0103-7) contains supplementary material, which is available to authorized users.

## Background

Metabolic syndrome (MetS), defined by clustering of overweight (obesity), elevated blood pressure, impaired glucose metabolism, dyslipidemia and typically characterized by insulin resistance, has become one of the major public health challenges worldwide [1–4]. Although the clinical value of MetS has controversy, there is agreement that clustering of risk factors predicts an increased risk of developing cardiovascular disease and helps identifying individuals at high risk of both type 2 diabetes and CVD [1, 5–7]. Fibrinogen, a key plasma protein synthesized in the liver, plays an important role in coagulation, plasma viscosity and erythrocyte aggregation [8]. Thus, elevated levels of fibrinogen contribute to a prothrombotic state, and may play an important role in the development of MetS [9, 10].

Epidemiologically, several cross-sectional studies had suggested that fibrinogen was possibly linked with MetS/its components [11–17]. Specifically, a cross-sectional study in British birth cohort population showed a stronger correlation in females than in males, indicating the differences between genders [14]. While, a cohort study in Turkish adults reported a reverse conclusion with significant association in males rather than in females [18]. Furthermore, another prospective cohort study in Framingham offspring study attempted to identify the association, but found no relationship between them [19]. Therefore, considering these controversial results, it was of great value to identify the association of fibrinogen with MetS, and further to clarify what role of fibrinogen played in the development of MetS.

To achieve above purposes, we conducted a large-scale cohort study in urban Han Chinese population. The original cohort was firstly constructed from routine health check-up population from 2005 to 2011without MetS at baseline. Then, 4 pre-MetS sub-cohorts (overweight, dyslipidemia, hypertension and hyperglycemia), were also created from the original cohort, with all overweight, dyslipidemia, hypertension and hyperglycemia subjects at baseline included respectively. Various strategies of Cox model analysis were performed for attempting to confirm the role of fibrinogen in the development of MetS.

## Methods

### Study population and cohort design

The cohort study was conducted in routine health check-up population at Center for Health Management of Shandong Provincial Qianfoshan Hospital and Shandong Provincial Hospital. The original cohort was constructed from this routine health check-up population from 2005 to 2011, and its framework was showed in Additional file 1: Figure S1. A total of 6209 participants free of MetS and other cardiovascular diseases were included in this cohort, to follow up for MetS with an annually regular clinical and laboratory examination. All subjects were more than 40-year-old, with 3843 males and 2366 females respectively. If necessary, 4 pre-MetS sub-cohorts, with overweight, dyslipidemia, hypertension and hyperglycemia at baseline included respectively, would be created for further exploring the role of fibrinogen in the development of MetS. The study protocol was approved by ethics committee of School of Public Health, Shandong University. Written informed consent was obtained from all participants.

### Measurements

At baseline, plasma fibrinogen was tested by Clauss method [20]. MetS related biomarkers, including systolic blood pressure, diastolic blood pressure, body mass index, fasting plasma glucose (FPG), total cholesterol, triglyceride, HDL-cholesterol, LDL-cholesterol, were measured annually with standard clinical and laboratory protocol for follow-up of MetS. Lifestyle factors, including smoking, drinking, vegetarian diet and exercise, were also obtained using standardized questionnaire. According to self-report smoking condition, smoking was categorized into never, former and current smoker. Subjects who reported frequently drinking were defined as frequent drinker; all others were non-frequent drinker. Subjects who took plant-based foods (mainly plant, but also took some meat) were defined as vegetarian; all others were non-vegetarian. Subjects who took more than 5 times 30 min of moderate activity per week or more than 3 times 20 min of vigorous activity per week, or equivalent were defined as regular exerciser; all others were non-regular exerciser.

### Diagnosis of MetS

The MetS was diagnosed using criteria of Chinese medical association diabetes branch (CDS) [21], defined as meeting three or more of the following disorders: (1) overweight or overweight (BMI ≥ 25.0 kg/M^2^); (2) hypertension (SBP ≥ 140 mmHg, DBP ≥ 90 mmHg or diagnosed before); (3) hyperglycemia (FPG ≥ 6.1 mmol/L or 2 h Postmeal Glucose ≥7.8 mmol/L, or diagnosed before); (4) dyslipidemia (TG ≥ 1.7 mmol/L, or HDL < 0.9 mmol/L in male and <1.0 mmol/L in female).

### Statistical analysis

To account for missing values of covariates, multiple imputations were performed, and the Markov chain Monte Carlo (MCMC) method was chosen according to MI Procedure of SAS [22]. Most variables had less than 2 % missing observations before imputation except smoking, drinking, diet and physical activity having less than 10 % missing values.

Continuous variables were presented by mean (standard deviation) and categorical variables were summarized as percentages. The fibrinogen level, originally continuous, was categorized into four quartiles (Q1–Q4), with P25, P50 and P75 as cut-off values. Participants with and without incident MetS during follow-up was compared using *t* test for continuous variables, and Chi square test for categorical variables. Chi square trend test was used to detect the trend of MetS incidence with increasing of fibrinogen from Q1 to Q4. Cox proportional hazards model was further used to detect the association of fibrinogen with MetS after adjusting potential confounders, including age, smoking, drinking, vegetarian diet and exercise, as well as baseline status of overweight, dyslipidemia, hypertension and hyperglycemia. Further analysis was conducted in four sub-cohorts (overweight, dyslipidemia, hypertension and hyperglycemia). Specifically, to detect whether fibrinogen was interacted with overweight on MetS, fibrinogen × overweight was put into the Cox model with adjusting other potential confounders in original female cohort; and to further illustrate the role of fibrinogen in the pathway from overweight to MetS, stratification analysis with Cox model was conducted in the 4 sub-groups of fibrinogen quartiles. All data analysis was performed using SAS version 9.2 (SAS Institute, Inc., Cary, NC, USA). A two-sided P < 0.05 was considered to be statistically significant.

## Results

In this cohort, among 6209 (3843 males and 2366 females) participants free of MetS at baseline, 1175 MetS (853 males and 322 females) were observed during the 5-year follow-up, and the total incidence density was up to 75.58 per 1000 person-years (1175/15547), with 89.85 (853/9494) and 53.20 per 1000 person-years (322/6053) for male and female respectively.

Table 1 showed the baseline fibrinogen and MetS related factors in MetS and non-MetS within total, male and female groups. Obviously, fibrinogen level was significantly higher in MetS than in non-MetS for entire cohort (t value = −4.39, P < 0.05) and for both male (t value = −2.62, P < 0.05) and female (t value = −5.98, P < 0.05), and significant differences of other factors, except smoking in male and smoking, drinking, vegetarian diet in female, were also detected between MetS and non-MetS in the two groups. In addition, baseline characteristics of participants grouped by fibrinogen quartiles were also showed in Additional file 2: Table S1.

**Table 1:** Characteristics comparison between participants with and without incident MetS during follow-up in total, males and females

Characteristics	Total			Males			Females		
	Non-MetS (n = 5034)	MetS	P value	Non-MetS (n = 2990)	MetS	P value	Non-MetS (n = 2044)	MetS (n = 322)	P value
		(n = 1175)			(n = 853)				
Fibrinogen (g/L)	3.29 ± 0.81	3.4 ± 0.83	<0.0001	3.21 ± 0.8	3.30 ± 0.82	0.0088	3.39 ± 0.81	3.68 ± 0.81	<0.0001
Age	51.55 ± 10.2	55.29 ± 11.2	<0.0001	52.03 ± 10.57	53.98 ± 11.00	<0.0001	50.84 ± 9.60	58.78 ± 10.99	<0.0001
Male (%)	2990 (59.4)	853 (72.6)	<0.0001						
Female (%)	2044 (40.6)	322 (27.4)	<0.0001						
Overweight (%)	1853 (36.81)	816 (69.45)	<0.0001	1334 (44.62)	618 (72.45)	<0.0001	519 (25.39)	198 (61.49)	<0.0001
Dyslipidemia (%)	1352 (26.86)	444 (37.79)	<0.0001	969 (32.41)	344 (40.33)	<0.0001	383 (18.74)	100 (31.06)	<0.0001
Hypertension (%)	977 (19.41)	495 (42.13)	<0.0001	629 (21.04)	332 (38.92)	<0.0001	348 (17.03)	163 (50.62)	<0.0001
Hyperglycemia (%)	230 (4.57)	136 (11.57)	<0.0001	155 (5.18)	96 (11.25)	<0.0001	75 (3.67)	40 (12.42)	<0.0001
Former smoker	164 (3.26)	50 (4.26)	0.008	122 (4.08)	48 (5.63)	0.1125	42 (2.05)	2 (0.62)	0.1893
Current smoker (%)	1430 (28.41)	375 (31.91)	0.008	1331 (44.52)	361 (42.32)	0.1125	99 (4.84)	14 (4.35)	0.1893
Frequent drinker (%)	1189 (23.62)	354 (30.13)	<0.0001	1070 (35.79)	339 (39.74)	0.0344	119 (5.82)	15 (4.66)	0.4012
Vegetarian (%)	1535 (30.49)	296 (25.19)	0.0003	745 (24.92)	177 (20.75)	0.012	790 (38.65)	119 (36.96)	0.5615
Regular exercise (%)	2860 (56.81)	583 (49.62)	<0.0001	1599 (53.48)	418 (49.00)	0.021	1261 (61.69)	165 (51.24)	0.0004
BMI (kg/m^2^)	24.18 ± 4.55	26.5 ± 2.74	<0.0001	24.80 ± 2.70	26.67 ± 2.61	<0.0001	23.27 ± 6.23	26.04 ± 3.01	<0.0001
Systolic BP (mmHg)	122.75 ± 17.71	134.35 ± 17.8	<0.0001	124.97 ± 16.84	133.74 ± 17.16	<0.0001	119.49 ± 18.43	135.96 ± 19.3	<0.0001
Diastolic BP (mmHg)	73.08 ± 10.48	79.32 ± 10.57	<0.0001	74.77 ± 10.25	79.91 ± 10.26	<0.0001	70.6 ± 10.31	77.77 ± 11.21	<0.0001
FPG (mg/dl)	5.05 ± 0.75	5.5 ± 1.07	<0.0001	5.09 ± 0.76	5.48 ± 1.05	<0.0001	4.98 ± 0.73	5.54 ± 1.11	<0.0001
Total cholesterol (mg/dl)	5.19 ± 0.92	5.36 ± 1	<0.0001	5.14 ± 0.88	5.23 ± 0.94	0.0103	5.26 ± 0.98	5.71 ± 1.07	<0.0001
Triglyceride (mg/dl)	1.37 ± 0.99	1.79 ± 1.33	<0.0001	1.53 ± 1.08	1.85 ± 1.46	<0.0001	1.13 ± 0.78	1.62 ± 0.92	<0.0001
HDL-cholesterol (mg/dl)	1.35 ± 0.32	1.26 ± 0.29	<0.0001	1.26 ± 0.28	1.23 ± 0.30	0.0012	1.47 ± 0.32	1.35 ± 0.26	<0.0001
LDL-cholesterol (mg/dl)	2.95 ± 0.71	3.11 ± 0.72	<0.0001	3.00 ± 0.69	3.06 ± 0.71	0.0208	2.88 ± 0.73	3.25 ± 0.73	<0.0001

**Fig. 1:**
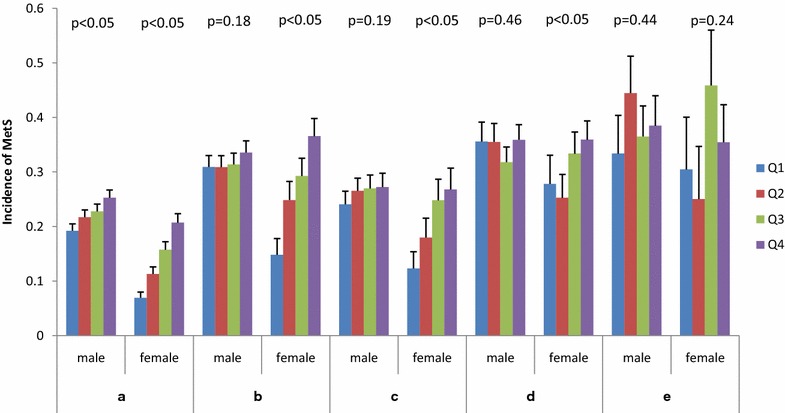
The incidence of MetS by quartiles of fibrinogen in whole participants (**a**) and in overweight (**b**), dyslipidemia (**c**), hypertension (**d**), and hyperglycemia (**e**) sub-cohorts in male and female. In whole participants (**a**), 853 of 3843 males and 322 of 2366 females developed MetS during follow-up; In overweight sub-cohort (**b**), 618 of 1952 males and 198 of 717 females developed MetS during follow-up; In dyslipidemia sub-cohort (**c**), 344 of 1313 males and 100 of 483 females developed MetS during follow-up; In hypertension sub-cohort (**d**), 332 of 961 males and 163 of 511 females developed MetS during follow-up; In hyperglycemia sub-cohort (**e**), 96 of 251 males and of 115 females developed MetS during follow-up

Figure 1 illustrated the incidence of MetS by quartiles of fibrinogen in the whole participants (a), as well as in the overweight (b), dyslipidemia (c), hypertension (d), and hyperglycemia (e) sub-cohorts for male and female group respectively. For the whole participants, an obvious increasing trend of MetS incidence with fibrinogen was observed in both male (chisq = −3.2073, *P* = 0.0007) and female (chisq = −7.2832, P < 0.0001). However, the increasing slope was much stronger in female than in male, especially in overweight and dyslipidemia sub-cohorts. Specifically, in the four sub-cohorts of female, increasing trends of MetS incidence with fibrinogen were observed in overweight (chisq = −4.5632, P < 0.0001), dyslipidemia (chisq = −3.0322, *P* = 0.0012), and in hypertension (chisq = −1.8330, *P* = 0.0334), while no increasing trend revealed in hyperglycemia (chisq = −0.7136, *P* = 0.2377). In contrary, no increasing trends were found in the four male sub-cohorts.

Table 2 illustrated the RR (95 % CIs) from Cox models with different adjusting factors in the entire cohort as well as in male and female respectively. In female, model 1 and model 2 suggested an obviously significant dose–response effect of fibrinogen on MetS with Q1 (fibrinogen ≤2.92 g/L) as reference level, with P value for trend of 0.0011 and 0.0498 for model 1 and model 2 respectively. By adjusting age, smoking, drinking, vegetarian diet and exercise (model 1), the RR (95 % CI) of fibrinogen on MetS (Q4 vs. Q1) was 1.84 (1.27, 2.66). After further adjusting the four MetS components (overweight, dyslipidemia, hypertension, hyperglycemia) in model 2, the RR became 1.48 (1.02, 2.13), indicating that fibrinogen might be an independent risk factor of MetS in females. However, similar trend was not observed in male as well as in the entire cohort.

**Table 2:** Risk ratios (RR) and their 95 % confidence intervals (CI) from Cox model for association of fibrinogen on MetS in male and female

	Total (n = 6209)		Male (n = 3637)		Female (n = 2260)		
	Model 1	Model 2	Model 1	Model 2	Model 1	Model 2	Model 3
Fibrinogen quartiles							
Q2 vs. Q1	1.12 (0.94,1.33)	1.03 (0.87,1.23)	1.09 (0.89,1.32)	1.00 (0.82,1.22)	1.43 (0.97,2.12)	1.33 (0.90,1.96)	1.04 (0.58,1.87)
Q3 vs. Q1	1.19 (1.01,1.41)	1.01 (0.85,1.20)	1.11 (0.91,1.35)	0.92 (0.76,1.12)	1.66 (1.14,2.4)	1.45 (1.00,2.10)	1.22 (0.71,2.10)
Q4 vs. Q1	1.39 (1.17,1.65)	1.15 (0.97,1.36)	1.22 (1,1.48)	1.05 (0.86,1.28)	1.84 (1.27,2.66)	1.48 (1.02,2.13)	1.03 (0.60,1.77)
*P value for trend*	0.0019	0.1925	0.0523	0.8046	0.0011	0.0498	
Age	1.03 (1.02,1.03)	1.02 (1.01,1.02)	1.02 (1.01,1.02)	1.01 (1.00,1.02)	1.05 (1.04,1.06)	1.03 (1.02,1.04)	1.03 (1.02,1.04)
Female	0.65 (0.57,0.75)	0.89 (0.77,1.03)					
Former smoking	1.06 (0.79,1.42)	1.22 (0.91,1.64)	1.18 (0.87,1.59)	1.32 (0.98,1.79)	0.39 (0.09,1.61)	0.45 (0.11,1.88)	0.46 (0.11,1.94)
Current smoking	0.95 (0.83,1.09)	0.99 (0.86,1.14)	0.93 (0.81,1.08)	0.98 (0.85,1.14)	0.87 (0.49,1.52)	0.96 (0.55,1.67)	0.96 (0.55,1.68)
Frequent drinker	1.34 (1.17,1.55)	1.19 (1.03,1.37)	1.3 (1.12,1.51)	1.18 (1.02,1.37)	0.99 (0.57,1.72)	0.96 (0.55,1.67)	0.95 (0.54,1.65)
Vegetarian	0.77 (0.68,0.89)	0.83 (0.72,0.95)	0.78 (0.65,0.92)	0.83 (0.70,0.99)	0.77 (0.61,0.97)	0.81 (0.64,1.02)	0.81 (0.64,1.02)
Regular exerciser	1.03 (0.92,1.16)	1.02 (0.91,1.15)	1.02 (0.89,1.17)	1.02 (0.89,1.17)	1.17 (0.93,1.46)	1.06 (0.84,1.32)	1.04 (0.83,1.31)
Overweight		3.60 (3.15,4.11)		3.29 (2.81,3.87)		4.11 (3.23,5.22)	2.76 (1.5,5.06)
Dyslipidemia		2.36 (2.06,2.71)		2.13 (1.81,2.50)		2.75 (2.11,3.57)	2.82 (2.16,3.67)
Hypertension		3.04 (2.64,3.5)		2.83 (2.39,3.35)		3.20 (2.46,4.18)	3.26 (2.49,4.25)
Hyperglycemia		3.48 (2.87,4.23)		3.26 (2.59,4.11)		3.85 (2.70,5.48)	3.95 (2.76,5.63)
Overweight*fibrinogen							
1*Q2							1.54 (0.70,3.38)
1*Q3							1.37 (0.66,2.88)
1*Q4							1.85 (0.90,3.80)

For fibrinogen quartiles, in whole participates, Q1 was defined as fibrinogen ≤2.79 g/l, Q2 was defined as fibrinogen in 2.79–3.20 g/l, Q3 was defined as fibrinogen in 3.20–3.567 g/l, and Q4 was defined as fibrinogen >3.67 g/l; in male, Q1 was defined as fibrinogen ≤2.71 g/l, Q2 was defined as fibrinogen in 2.71–3.14 g/l, Q3 was defined as fibrinogen in 3.14–3.58 g/l, and Q4 was defined as fibrinogen >3.58 g/l; in female, Q1 was defined as fibrinogen ≤2.92 g/l, Q2 was defined as fibrinogen in 2.92–3.32 g/l, Q3 was defined as fibrinogen in 3.32–3.81 g/l, and Q4 was defined as fibrinogen >3.81 g/l

Model 1. adjusted by baseline covariates of age, gender, smoking, alcohol, vegetarian diet, exercise

Model 2. adjusted by baseline covariates of age, gender, smoking, alcohol, vegetarian diet, exercise, overweight, hypertension, hyperglycemia, dyslipidemia

Model 3. adjusted by baseline covariates of age, gender, smoking, alcohol, vegetarian diet, exercise, overweight, hypertension, hyperglycemia, dyslipidemia, interaction of fibrinogen and overweight

As fibrinogen associated with MetS just in female, 4 pre-MetS female sub-cohorts, with overweight (n = 717), dyslipidemia (n = 483), hypertension (n = 511) and hyperglycemia (n = 115) at baseline, was created for further exploring the role of fibrinogen in the development of MetS. Table 3 showed their RR (95 % CI) from Cox models with adjusting other potential confounders. Obviously, significant association of fibrinogen with MetS was only detected in the overweight sub-cohort, with RR of 1.97(95 % CI 1.20, 3.23) for Q4 vs. Q1.

**Table 3:** Risk ratios (RR) and their 95 % confidence intervals (CI) from Cox model for association of fibrinogen on MetS in female overweight, dyslipidemia, hypertension and hyperglycemia sub-cohorts

	Overweight (n = 717)	Dyslipidemia (n = 483)	Hypertension (n = 511)	Hyperglycemia (n = 115)
Fibrinogen quartiles				
Q2 vs. Q1	1.67 (0.98,2.84)	1.28 (0.65,2.54)	0.90 (0.50,1.62)	0.77 (0.24,2.51)
Q3 vs. Q1	1.69 (1.02,2.81)	1.49 (0.78,2.84)	1.07 (0.63,1.84)	1.26 (0.46,3.43)
Q4 vs. Q1	1.97 (1.20,3.23)	1.44 (0.75,2.76)	1.10 (0.65,1.85)	0.77 (0.30,1.97)
Age	1.03 (1.02,1.05)	1.04 (1.02,1.07)	1.02 (1.00,1.03)	1.03 (1.00,1.07)
Smoking	0.92 (0.63,1.35)	1.25 (0.75,2.07)	1.00 (0.67,1.47)	1.60 (0.71,3.59)
Frequent drinker	0.72 (0.33,1.58)	0.38 (0.11,1.39)	0.97 (0.48,1.97)	3.24 (0.69,15.1)
Vegetarian	0.78 (0.57,1.05)	0.98 (0.65,1.49)	0.82 (0.59,1.13)	0.76 (0.37,1.56)
Regular exerciser	0.77 (0.57,1.03)	0.82 (0.55,1.22)	1.08 (0.79,1.48)	1.02 (0.52,2.01)
Overweight		4.34 (2.52,7.47)	4.18 (2.63,6.66)	2.89 (1.07,7.83)
Dyslipidemia	2.59 (1.70,3.94)		3.26 (1.87,5.69)	1.64 (0.52,5.17)
Hypertension	2.94 (1.98,4.34)	3.49 (1.89,6.45)		2.22 (0.79,6.24)
Hyperglycemia	3.29 (1.75,6.18)	3.16 (1.25,7.98)	4.09 (2.11,7.95)	

As fibrinogen was associated with MetS just in overweight sub-cohort of females, the interaction of fibrinogen and overweight should be detected in the original female cohort. After adjusting age, smoking, drinking, vegetarian diet, exercise, hypertension, hyperglycemia, dyslipidemia, no statistically significant interaction of overweight and fibrinogen on MetS was revealed, and the main effect of overweight was still statistically significant whilst fibrinogen non-significant (Seeing model 3 in Table 2), suggesting that fibrinogen might be a modifier in the pathway from overweight to MetS.

To illustrate whether fibrinogen was a modifier in the pathway from overweight to MetS, stratification analysis with Cox model was conducted in the 4 sub-groups of fibrinogen quartiles. As showed in Table 4, effects of overweight on MetS were quite different among the four sub-groups, with RR of 2.98 for Q1, 4.40 for Q2, 3.93 for Q3, and 4.82 for Q4 respectively, indicating that fibrinogen was a potential modifier in the pathway from overweight to MetS.

**Table 4:** Risk ratios (RR) and their 95 % confidence intervals (CI) from Cox model for different effects of overweight on MetS in 4 sub-groups of fibrinogen quartiles in female cohort

	Q1 (≤2.79 g/l)	Q2 (2.79–3.20 g/l)	Q3 (3.20–3.67 g/l)	Q4 (>3.67 g/l)
	(n = 607)	(n = 577)	(n = 592)	(n = 590)
Age	1.04 (1.01,1.08)	1.04 (1.01,1.06)	1.02 (1.00,1.05)	1.03 (1.01,1.05)
Smoking	1.06 (0.48,2.35)	0.36 (0.11,1.20)	1.01 (0.64,1.61)	1.21 (0.78,1.89)
Frequent drinker	0.44 (0.06,3.42)	1.77 (0.49,6.30)	0.77 (0.30,1.98)	1.01 (0.41,2.45)
Vegetarian	0.89 (0.46,1.7)	1.16 (0.69,1.95)	0.69 (0.45,1.08)	0.74 (0.51,1.09)
Regular exerciser	0.96 (0.51,1.84)	1.14 (0.68,1.90)	1.01 (0.66,1.54)	1.03 (0.71,1.48)
*Overweight*	*2.98 (1.56,5.7)*	*4.40 (2.60,7.44)*	*3.93 (2.51,6.15)*	*4.82 (3.16,7.35)*
Dyslipidemia	3.28 (1.62,6.64)	2.21 (1.27,3.84)	3.01 (1.84,4.92)	2.77 (1.75,4.38)
Hypertension	5.36 (2.66,10.79)	2.50 (1.41,4.46)	3.59 (2.15,5.98)	3.06 (1.96,4.77)
Hyperglycemia	4.96 (2.01,12.23)	3.24 (1.24,8.45)	5.75 (2.93,11.31)	3.09 (1.76,5.42)

## Discussion

In this cohort study, the total MetS incidence density of 75.58 per 1000 person-years observed in this specific urban Han Chinese population during the 5-year follow-up, with 89.85 and 53.20 for male and female respectively, and statistically significant difference of fibrinogen level between MetS and non-MetS groups was further detected in the two gender groups (Table 1). Similar gender difference of MetS was also reported in British [14], Pakistan [23], Frinks cohort [24], and TLGS cohort [25] population. In the contrary, female dominance of MetS was also reported in several populations [26–29]. The heterogeneity of MetS between genders among different population might be due to their different risk factor exposure pattern, suggesting that gender stratification analysis should be considered for further detecting the association of fibrinogen with MetS.

A much stronger increasing trend of MetS incidence with fibrinogen quartiles was observed in female rather than in male (Fig. 1a). Specifically, similar obviously increasing trends were also detected in overweight, dyslipidemia, and hypertension female sub-cohorts (Fig. 1b–d). Further Cox regression analysis by adjusting for baseline smoking, exercise and the four MetS components showed an obviously significant association of fibrinogen with MetS in female, but no association was detected in male (Table 2). Similar gender difference was also reported in British population by cross-sectional study in British birth cohort [14] while reverse trend was claimed in Turkish cohort study [18]. These gender differences in different population remained a controversial issue for further investigations. One possible explanation might be that in the development of MetS, fibrinogen, as a proinflammatory factor, acted largely addictively to insulin resistance in female, whereas such effect might not exist in male [30–32].

At present study, fibrinogen associated with MetS just in female reminded us to focus on the association analysis in overweight, dyslipidemia, hypertension and hyperglycemia female subjects respectively. As showed in Table 3, significantly positive association was only revealed in overweight sub-cohort. This indicated that fibrinogen played a major role in the pathway from overweight to MetS for female. A perceptive review, about obesity, haemostasis and the fibrinolytic system [33], provided clues for hunting the role of fibrinogen in this pathway. The authors highlighted the pathway from insulin resistance/hyperinsulinaemia to obesity to traditional MetS components (hypertension, hyperglycemia and dyslipidemia) and haemostatic/fibrinolytic disturbances, and finally to cardiovascular diseases; and further concluded that fibrinogen, as a downstream component of the inflammatory cascade [34, 35] in the pathway, was increased in overweight (obese) subjects, and associated with insulin resistance. However, from this review, what role of fibrinogen played in the pathway of overweight to MetS still remained unclear. For fibrinogen positively associated with MetS just in female, it might be partly explained by the effect of estrogen. A cross-sectional study [36] reported that fibrinogen was positively correlated with endogenous estrone, while a cohort study [37] further reported that estradiol was positively associated of plasma fibrinogen, especially in subjects who were overweight.

To detect whether fibrinogen was interacted with overweight on MetS, fibrinogen*overweight was put into the Cox model with adjusting other potential confounders (Showed in model 3, Table 2). Unexpectedly, although the main effect of overweight still statistically significant, no significant interaction of overweight*fibrinogen on MetS was revealed, and the main effect of fibrinogen became non-significant simultaneously. The results suggested that fibrinogen might be a potential modifier in the pathway from overweight to MetS. This hypothesis was supported by our further stratification analysis in the 4 sub-groups of fibrinogen quartiles with Cox model by adjusting other potential confounders. Because effects of overweight on MetS were quite different among the 4 sub-groups of fibrinogen quartiles, with RR of 2.98 for Q1, 4.40 for Q2, 3.93 for Q3, and 4.82 for Q4 respectively (showed in Table 4). These suggested that female individuals with overweight and elevated fibrinogen should pay more attention to their blood pressure, blood sugar and blood lipid for early prevention of MetS.

In fact, the mechanism of fibrinogen in the development of MetS was extremely complex. Statistically, we found that fibrinogen might promote individuals with overweight to develop into MetS. But their causal relationship still remained uncertain. It is possible that fibrinogen might be an accompanying biomarker in the development of MetS from overweight. Therefore, further experimental researches should be conducted for confirming the role of fibrinogen in the development of MetS.

Selection bias and information bias were inevitable in this cohort study, due to its biased subjects from routine health check-up in this specific urban Han Chinese population, and relatively shorter follow-up period. In addition, for only BMI was used to measure overweight in health check-up setting, the diagnostic criteria of MetS was based on China Diabetes Federation, rather than international standard criteria. CRP and other inflammation biomarkers were not measured, so associations for additional biomarkers and adjustment for CRP could not be conducted in this study. Further unbiased investigations with long follow-up period should be conducted in general community population.

## Conclusions

Plasma fibrinogen was associated with MetS just in overweight sub-cohort of female individuals, and it might be a potential modifier in the pathway from overweight to MetS.

## Additional files

10.1186/s13098-015-0103-7 The framework of original cohort.
10.1186/s13098-015-0103-7 Baseline characteristics of participants grouped by fibrinogen quartiles.

## Abbreviations

MetS: metabolic syndrome
CI: confidence interval
RR: risk ratio
