# Supplementary figures and images for: A new insight into the role of plasma fibrinogen in the development of metabolic syndrome from a prospective cohort study in urban Han Chinese population
Source: Diabetol Metab Syndr. 2015 Dec 2;7:110. doi: 10.1186/s13098-015-0103-7 (PMC4667450; doi:10.1186/s13098-015-0103-7)

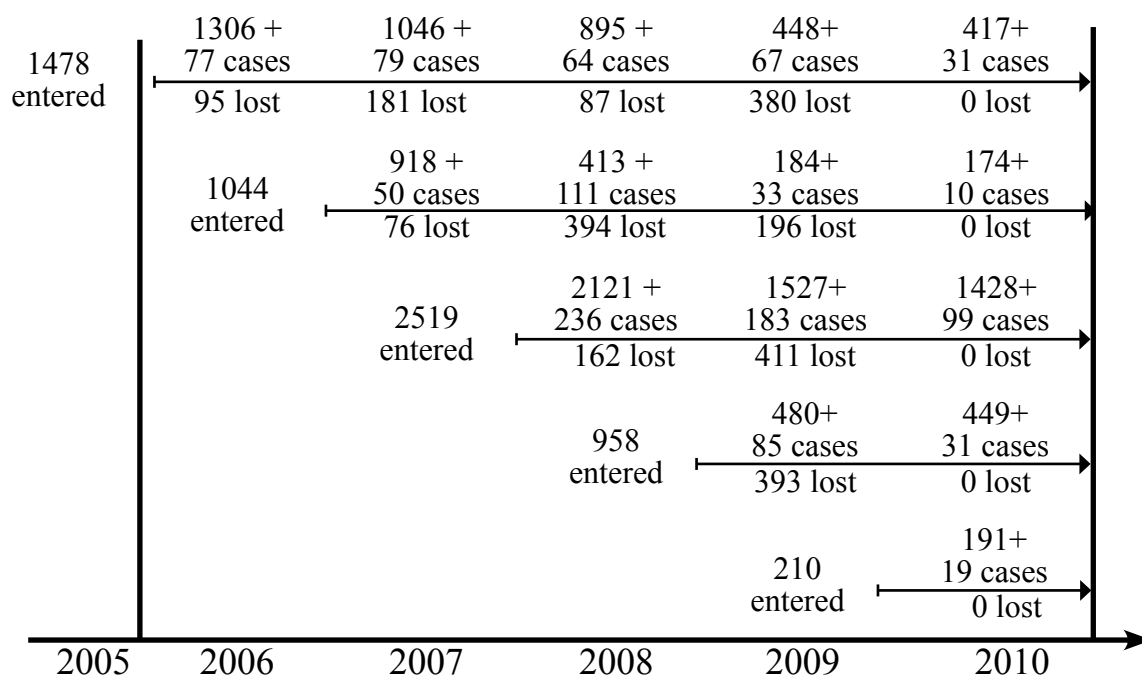

Figure S1. The framework of original cohort.

Supplement: Supplementary file 1 — 10.1186/s13098-015-0103-7 The framework of original cohort. [file 13098_2015_103_MOESM1_ESM.pdf]
