# Supplementary material for: A new insight into the role of plasma fibrinogen in the development of metabolic syndrome from a prospective cohort study in urban Han Chinese population
Source: Diabetol Metab Syndr. 2015 Dec 2;7:110. doi: 10.1186/s13098-015-0103-7 (PMC4667450; doi:10.1186/s13098-015-0103-7)
Supplement: Supplementary file 2 — 10.1186/s13098-015-0103-7 Baseline characteristics of participants grouped by fibrinogen quartiles. [file 13098_2015_103_MOESM2_ESM.docx]

Table S1. Baseline characteristics of participants grouped by fibrinogen quartiles.

| **Characteristics** | **Fibrinogen Quartiles, g/L** | | | | | |
| --- | --- | --- | --- | --- | --- | --- |
|  | **Q1 (≤2.79, n=1572)** | **Q2 (2.79-3.20, n=1538)** | **Q3 (3.20-3.67, n=1582)** | **Q4 (>3.67, n=1517)** | ***P value*** |  |
| Male (%) | 1120(71.25) | 980(63.72) | 938(59.29) | 805(53.07) | <0.0001 |  |
| Female (%) | 452(28.75) | 558(36.28) | 644(40.71) | 712(46.93) | <0.0001 |  |
| Age | 49.45±8.83 | 50.29±9.33 | 52.92±10.48 | 56.46±11.73 | <0.0001 |  |
| Obesity(%) | 652(41.48) | 635(41.29) | 712(45.01) | 670(44.17) | 0.0796 |  |
| Hypertension (%) | 261(16.6) | 301(19.57) | 423(26.74) | 487(32.1) | <0.0001 |  |
| Hyperglycemia (%) | 69(4.39) | 73(4.75) | 103(6.51) | 121(7.98) | <0.0001 |  |
| Hyperlipidemia (%) | 456(29.01) | 475(30.88) | 446(28.19) | 419(27.62) | 0.2082 |  |
| Number of baseline Mets components |  |  |  |  | <0.0001 |  |
| None | 583(37.09) | 511(33.22) | 461(29.14) | 396(26.1) |  |  |
| 1 | 540(34.35) | 570(37.06) | 558(35.27) | 545(35.93) |  |  |
| 2 | 449(28.56) | 457(29.71) | 563(35.59) | 576(37.97) |  |  |
| Current smoker (%) | 589(37.47) | 500(32.51) | 499(31.54) | 431(28.41) | <0.0001 |  |
| Regular exercise (%) | 924(58.78) | 884(57.48) | 837(52.91) | 798(52.6) | 0.0003 |  |
| BMI(kg/m2) | 24.44±2.96 | 24.34±7.05 | 24.86±2.97 | 24.84±2.99 | 0.0005 |  |
| Systolic BP (mmHg) | 121.79±16.14 | 123.33±16.75 | 126.08±19 | 128.66±20.3 | <0.0001 |  |
| Diastolic BP (mmHg) | 73.21±10.56 | 74.44±10.5 | 74.75±10.74 | 74.64±11.24 | 0.0001 |  |
| Fasting serum glucose (mg/dl) | 5.05±0.74 | 5.1±0.78 | 5.15±0.88 | 5.24±0.93 | <0.0001 |  |
| Total cholesterol (mg/dl) | 5.05±0.87 | 5.16±0.91 | 5.28±0.93 | 5.4±1.02 | <0.0001 |  |
| Triglyceride (mg/dl) | 1.46±1.13 | 1.46±1.19 | 1.43±0.98 | 1.45±0.99 | 0.8200 |  |
| HDL-cholesterol (mg/dl) | 1.33±0.31 | 1.33±0.33 | 1.33±0.31 | 1.33±0.3 | 0.8738 |  |
| LDL-cholesterol (mg/dl) | 2.87±0.68 | 2.93±0.68 | 3.02±0.71 | 3.11±0.77 | <0.0001 |  |

Data are expressed as means ± standard deviation for continuous variables, or frequency (percentages) for categorical variables.

* *P* values by one-way Anova for continuous variables and Chi square test for categorical variables.
